# Lactic acid inhibits melanin synthesis by regulating histone H3 lactylation and suppressing tyrp1 transcription in B16 melanoma cells

**DOI:** 10.1038/s41598-025-04225-8

**Published:** 2025-07-16

**Authors:** Bin Du, Meng Xiao, Liang Gao

**Affiliations:** 1 Shanxi Key Laboratory of Aging Mechanism Research and Translational Applications, Changzhi Medical College, Changzhi, 046000 China; 2 Department of Basic Medicine, Changzhi Medical College, Changzhi, 046000 China; 3 The Second School of Clinical Medicine, Changzhi Medical College, Changzhi, 046000 China

**Keywords:** Lactic acid, Melanin, Lactylation, Histone H3, Tyrp1, Post-translational modifications, Melanoma

## Abstract

Lactic acid, a widely distributed metabolic intermediate, exhibits superior permeability and mild acidity, making it a common reagent in skincare products. Lactic acid could significantly reduce melanin synthesis both in vitro and in vivo. However, the underlying molecular mechanisms remain less clear. To reveal the molecular mechanism by which lactic acid inhibits melanin synthesis through epigenetic pathways, proteins with significantly increased levels of lactylation modifications after lactic acid addition were identified through HPLC-MS/MS. Key genes affected by lactic acid addition were identified using ChIP-seq, and validated through qPCR and Western blot analyses. We reveal that lactic acid could promote protein lactylation in a dose-dependent manner in B16 cells. HPLC-MS analysis revealed that histone H3 showed the most significant increase in lactylation following lactic acid treatment. Through ChIP-seq analysis, we observed that lactylated histone H3 binding at the promoter region of the key melanogenesis gene, Tyrp1, significantly decreased after lactic acid treatment. This was accompanied by a notable reduction in Tyrp1 protein level. Our findings suggest that protein lactylation play a crucial role in lactic acid induced melanin synthesis inhibition.

**Supplementary Information:**

The online version contains supplementary material available at 10.1038/s41598-025-04225-8.

## Introduction

Lactic acid, a prevalent metabolic intermediate found in both nature and the human body^1^, is noted for its exceptional permeability and relatively mild acidity, which contribute to its frequent use in skincare formulations^2^.

Current research indicates that lactic acid may modulate melanin synthesis through multiple mechanisms. It has been demonstrated that lactic acid can directly inhibit melanin production by suppressing the activity of tyrosinase, the rate-limiting enzyme in the melanogenesis pathway^3^. Furthermore, lactic acid has the capacity to modify mitochondrial respiration and energy production^4^, resulting in decreased levels of intracellular reactive oxygen species (ROS)^5,6^, which are essential for melanin synthesis^7^. Despite these findings, the precise molecular mechanisms responsible for these effects remain insufficiently understood, highlighting the need for further investigation to comprehensively elucidate the involved pathways.

Furthermore, lactic acid plays a multifaceted role in biological systems, acting as a crucial mediator in cellular metabolism and signaling pathways. Its function transcends that of a mere metabolic byproduct, as it contributes to vital physiological processes such as pH regulation, energy production, and intracellular signaling, all of which are essential for maintaining cellular homeostasis and function^8^. These attributes render lactic acid a topic of considerable interest in dermatological research and therapeutic applications. Recent studies have demonstrated that elevated lactic acid levels can induce protein lactylation^9^, a novel post-translational modification. Lactylation modification occurs extensively in both histone and non-histone proteins. Histone lactylation (e.g., H3K18la) shares structural parallels with acetylation by neutralizing lysine charges, enhancing chromatin accessibility and facilitating gene expression through epigenetic regulation^10^. On the other hand, lactylation of non-histone proteins can directly modulate their functional properties. For example, lactylation of METTL3 significantly enhances its m6A methyltransferase activity, thereby regulating RNA methylation^11^. Conversely, lactylation of the tumor suppressor protein p53 inhibits its capacity to bind DNA containing p53 response elements, thereby facilitating tumor progression^12^. These findings underscore the diverse regulatory roles of lactylation in cellular processes and its potential implications for health and disease^13^.

Numerous studies have demonstrated that the administration of exogenous lactic acid to cells markedly increases intracellular protein lactylation levels^14^. This observation triggers the inquiry: does lactic acid supplementation inhibit pigment synthesis in melanocytes through alterations in protein lactylation? To address this question, we performed preliminary investigations utilizing proteomics and ChIP-seq. These techniques enabled us to examine alterations in protein lactylation and its potential impact on gene regulatory mechanisms associated with melanogenesis.

## Materials and methods

### Cell culture

Mouse melanoma B16-F10 cells were procured from Abcell. The cells were cultured in 1640 medium (C3010, Viva Cell) supplemented with 10% fetal bovine serum (164210-50, Procell) and 1% antibiotics. Passaging was performed when the cells reached 90% confluence.

### Melanin induction

To induce melanin production, B16 cells were initially passaged, and on the subsequent day, the culture medium was substituted with low-glucose DMEM (C3120, Viva Cell). Lactic acid (HY-Y0479, MCE) was then added at final concentrations of 5, 10, 15, and 20 mM, and the cells were incubated for either 24-48 h. Melanin accumulation within the cells was directly observed using microscopy. This experiment was conducted in triplicate.

### Detection of intracellular total melanin content

The cells were cultured in 6-well plate, and upon reaching the predetermined culture duration, they were harvested and subjected to centrifugation. The cells were then washed twice with PBS and resuspended in a solution of 1 M NaOH containing 10% DMSO. This mixture was incubated in a 65 °C water bath for 30 min to facilitate melanin dissolution. The melanin content was subsequently quantified by measuring absorbance at OD405 using a microplate reader.

### Immunofluorescence staining

During the cell culture process, cells were cultured on coverslips. Following the lactic acid treatment, cells were fixed using pre-cooled methanol for a duration of 10 min, follow by three times washes with PBS. Subsequently, the coverslips were blocked with 5% BSA for 1 h. After discarding the blocking solution, primary antibodies were directly applied and incubated overnight at 4 °C. On the following day, the coverslips underwent three additional PBS washes, each for 5 min. Secondary antibodie were then introduced, and the cells were incubated at room temperature for 1 h. Finally, the coverslips were mounted using a mounting medium containing DAPI and examined using an Olympus IX73 fluorescence microscope. The primary antibodies employed included anti-l-lactyl lysine rabbit monoclonal antibody, 1:1000 (PTM-1401RM, PTMBIO), anti-l-lactyl-histone H3 (Lys18) rabbit monoclonal antibody (PTM-1406RM, PTMBIO), and anti-TYRP1 rabbit monoclonal antibody (ab235447, Abcam).

### HPLC-MS

After culturing the cells to the designated time, they were lysed using SDT lysis buffer and digested via FASP. Lactic acid-modified peptides were enriched using a pan-lactylation antibody, desalted with a C18 cartridge, vacuum dried, and quantified for LC-MS analysis. Peptides were separated using an Easy-nLC 1200 nano-flow chromatography system (Thermo Scientific), and MS/MS analysis was performed. For each full scan, 20 precursor ions were selected for fragmentation, with MS2 resolution at 15,000 @m/z 200, AGC target: 1e5, Maximum injection time of 50 ms, HCD activation, an isolation window of 1.6 m/z, and normalized collision energy of 28. The raw LC-MS/MS data were processed using MaxQuant 2.0.1.0 for protein identification and quantification.

### ChIP-seq

B16 cells were treated with lactic acid at a final concentration of 20 mM for 48 h. Crosslinking was performed by adding an appropriate amount of formaldehyde to the cell culture medium. Subsequent experiments were carried out using the SimpleChIP^®^ Plus Sonication Chromatin IP Kit (56383, CST). Briefly, cell nuclei were isolated and extracted, followed by buffer exchange. DNA fragmentation was performed using a sonicator (M220, Covaris). Immunoprecipitation was conducted using Anti-L-Lactyl-Histone H3 (Lys18) Rabbit mAb (PTM-1406RM, PTMBIO). Sequencing was outsourced to Novogene Co., Ltd.

### qPCR

DNA isolated from the ChIP procedure was employed for quantitative PCR (qPCR) analysis.primers used are as follow:

Tyrp1-DNA-1-F: CTGTGGTGGGTACCCTGTGA

Tyrp1-DNA-1-R: GCTCTCGTGCACCCATATCC

Tyrp1-DNA-2-F: CTTCTCCCCAGCCCAAGAAT

Tyrp1-DNA-2-R: CCCACTCCGGAAATGGAATCT

### Western blot

Following cell harvest, lysis was performed using RIPA buffer supplemented with protease inhibitors. Protein concentrations were quantified via the BCA assay, after which the samples were diluted and subjected to heat denaturation. Proteins were resolved on a 10% SDS-PAGE gel and subsequently transferred onto a PVDF membrane employing a semi-dry transfer system. Post-transfer, the membrane was blocked with non-fat milk and incubated with the appropriate primary antibody overnight at 4 °C. The subsequent day, the membrane underwent washing with TBST and was incubated with an HRP-conjugated goat anti-rabbit secondary antibody at room temperature for 1 h. After additional TBST washes, an ECL reagent was applied, and the signal was detected using a gel imaging system.

### Statistical analysis

Statistical analyses were performed to evaluate the significance of differences among experimental groups. One-way analysis of variance (ANOVA) was used to compare the means of multiple groups. Data were expressed as mean ± standard deviation (SD), and a significance level of *p* < 0.05 was considered statistically significant. All analyses were conducted using GraphPad Prism 9.0.

### Statement

We hereby acknowledge that Deepseek R1 was employed for linguistic refinement and grammatical correction of this manuscript. The contributions of Deepseek R1 were confined to enhancing the clarity, coherence, and grammatical precision of the text. All intellectual content, ideas, and conclusions articulated in the paper remain exclusively the work of the authors. The utilization of AI tools did not affect the scientific integrity or originality of the research.

## Result

### Lactic acid inhibit melanin synthesis in a dose dependent manner

**Fig. 1:**
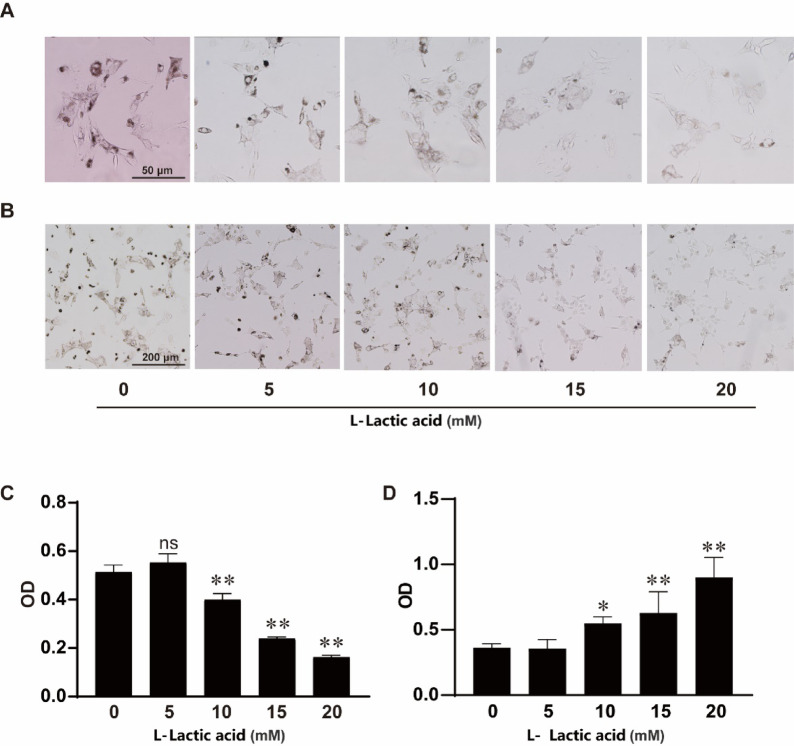
Lactic acid inhibits melanin production in B16 cell. (**A**,** B**) Intracellular melanin deposition in B16 cells, cells were cultured in DMEM (low glucose) for 48 h along with the treatment of 0, 5, 10 15, 20 mM lactic acid, with scale bars indicating 50 μm and 200 μm, respectively. (**C**) Melanin content was quantified by measuring optical density at 400 nm after dissolving intracellular melanin using 1 M NaOH containing 10% DMSO. (**D**) B16 cells were treated with lactic acid for 48 h, and cell viability was assessed using MTT analysis. *p < 0.05, **p < 0.01.

Lactic acid is known to reduce skin melanin production, though its molecular mechanisms are not fully understood. To investigate, B16 melanoma cells were treated with various lactic acid concentrations, revealing a significant decrease in intracellular melanin deposition with higher lactic acid levels, as shown in Fig. 1A–C. Cells were then dissolved using 1 M NaOH with 10% DMSO, confirming that lactic acid could inhibit melanin synthesis in B16 cells in a dose dependent manner. Lactic acid did not inhibit the viability of B16 cells, as illustrated in Fig. 1D.

### Lactic acid promote nuclear enrichment lactylation in a dose dependent manner

**Fig. 2:**
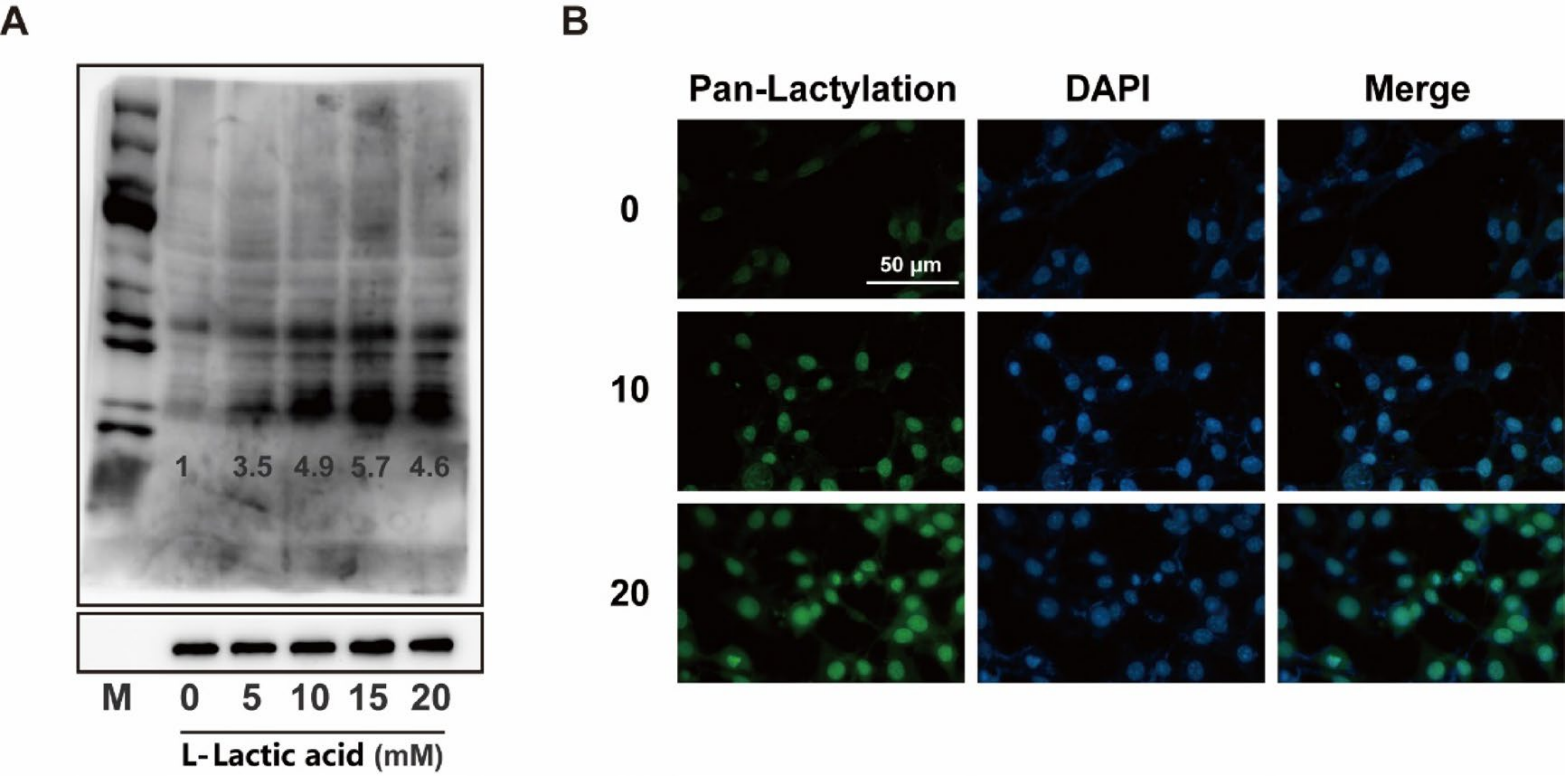
Lactic acid promotes pan-lactylation modifications. (**A**) Pan-lactylation levels in B16 cells treated with lactic acid at concentrations of 0–20 mM for 48 h. (**B**) Immunofluorescence analysis of lactylated proteins using anti-pan-lactylation antibody (green), showing subcellular localization of proteins with lactylation modifications. Scale bars indicate 50 μm.

To investigate the molecular mechanisms underlying the inhibition of melanin synthesis by lactic acid, we assessed the global levels of protein lactylation in cells exposed to lactic acid. Our findings demonstrated a significant, dose-dependent increase in total lactylation levels. Proteins with lactylation primarily distributed within the 15–45 kDa range (Fig. 2A). Immunofluorescence staining indicted that proteins with lactylation modifications were predominantly localized within the nucleus (Fig. 2B).

### Identification of proteins with lactylation via HPLC-MS/MS

**Fig. 3:**
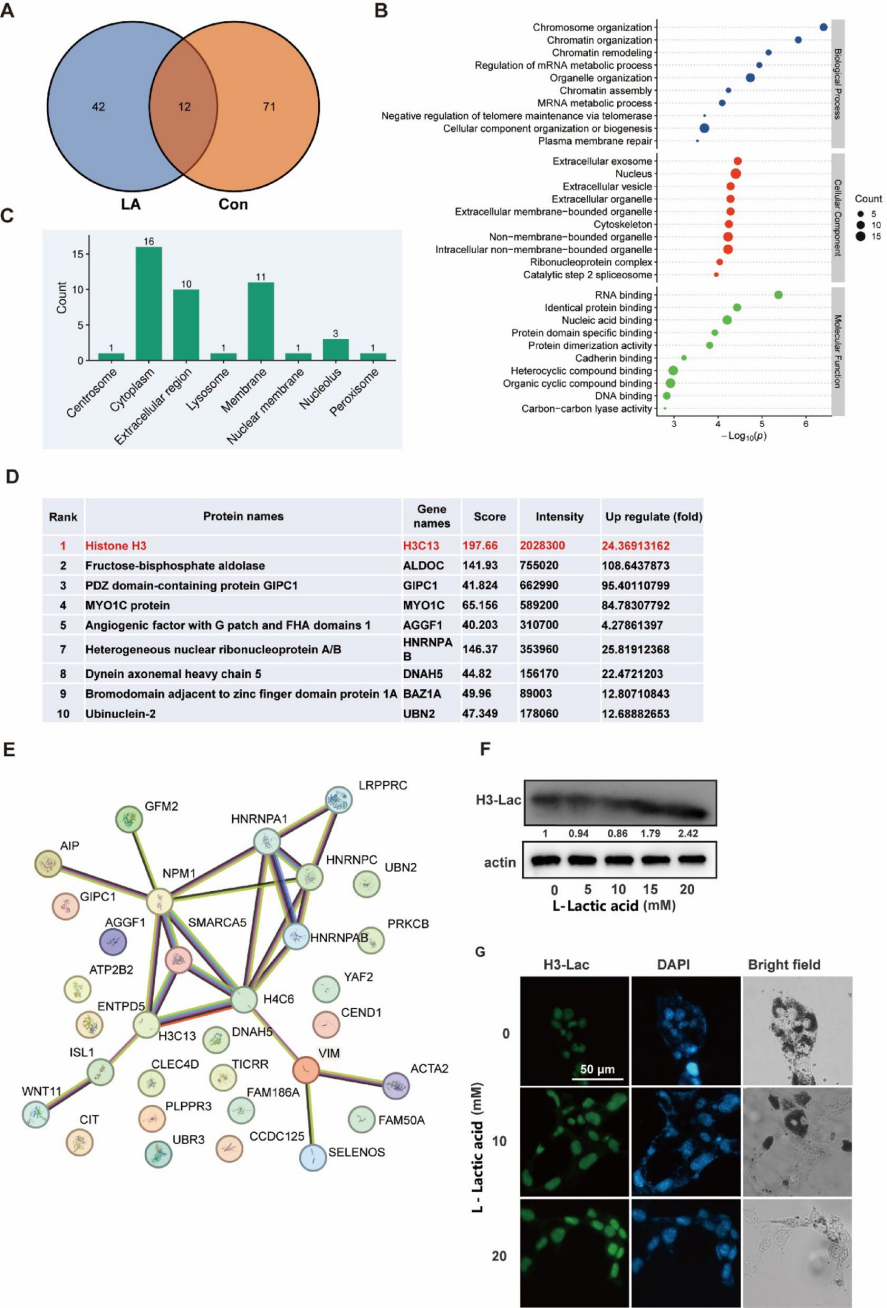
Lactic acid treatment significantly enhances lactylation of intracellular protein. (**A**) Venn diagram showing the differences in the number of lactylated proteins before and after lactic acid treatment, “Con” for control and “LA” for lactic acid; (**B**) GO analysis categorizing lactylated proteins into biological process, cellular component, and molecular function; (**C**) Top 10 proteins with elevated lactylation levels after lactic acid treatment; (**E**) Interaction network analysis of intracellular proteins with significantly altered lactylation modification; (**F**) Western blot analysis the lactylation levels of histone H3 after 0–20 mM lactic acid treatment for 48 h; (**G**) Immunofluorescence staining of histone H3 lactylation levels under different lactic acid concentrations, the scale bars represent 50 μm.

High-throughput mass spectrometry was employed to explore the variations in protein lactylation modifications caused by lactic acid treatment. The findings showed notable alterations in the proteins with lactylation in B16 cells exposed to 20 mM lactic acid for 48 h compared to those not treated (Fig. 3A). Gene Ontology (GO) analysis indicated that proteins with elevated lactylation levels in the lactic acid treatment group were mainly linked to biological processes like chromatin organization (Fig. 3B). Subcellular localization analysis revealed that lactylated proteins were extensively distributed across cellular compartments, such as the plasma membrane, centrosome, and cytoplasm (Fig. 3C).

Moreover, proteins exhibiting elevated levels of lactylation were ranked according to their intensity as detected by mass spectrometry, with histone H3 demonstrating the highest intensity (Fig. 3D). Additionally, STRING analysis indicated a significant enrichment of proteins associated with transcription and RNA splicing processes, such as H3C13, NPM1, and HNRNPA/B (Fig. 3E). Furthermore, we investigated the commonly reported histone H3 lactylation modification site, H3K18la. The increased H3Kla18 was validated through western blot analysis and immunofluorescence staining (Fig. 3F and G).

### ChIP-Seq analysis of histone H3 binding site alterations

**Fig. 4:**
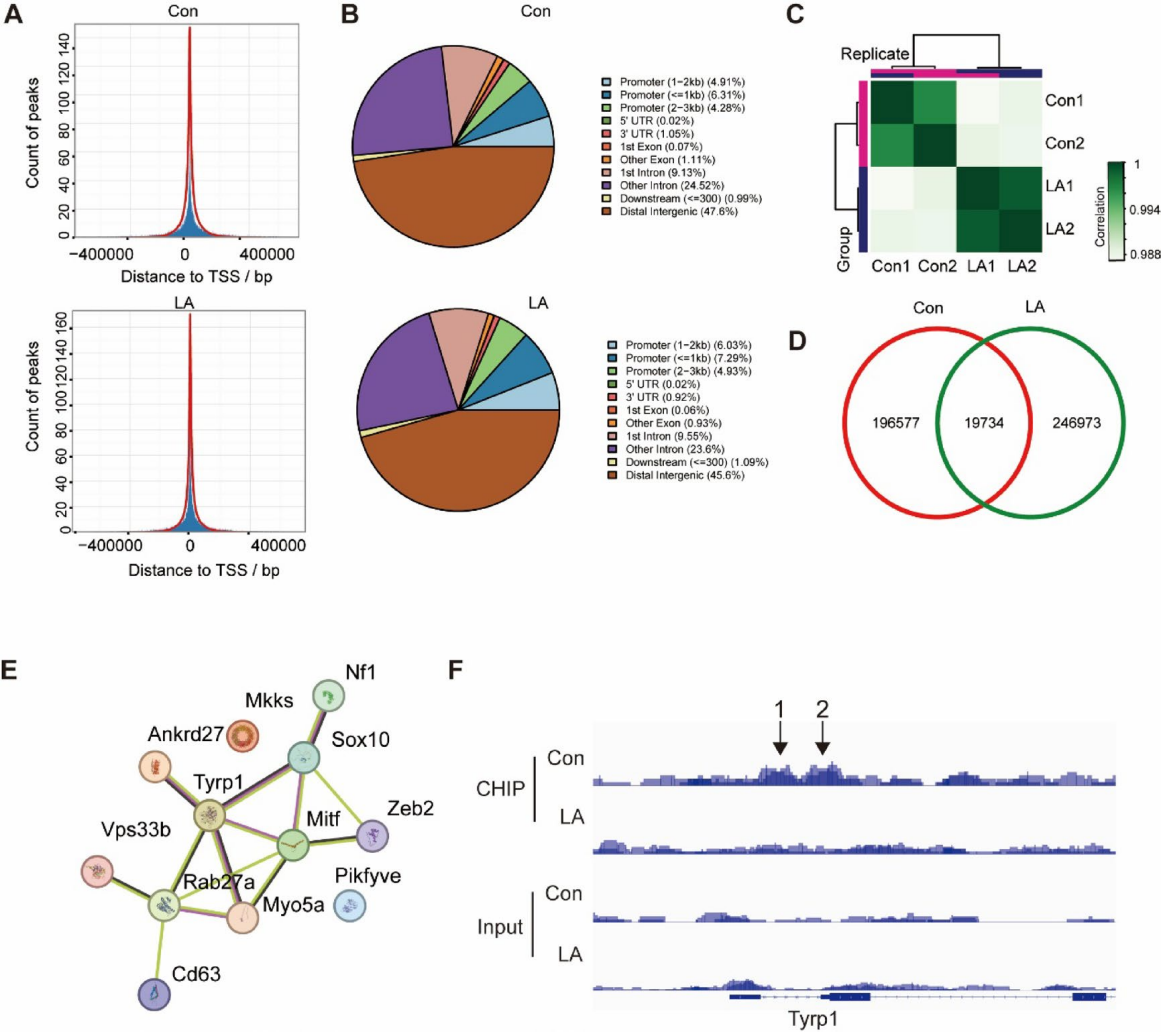
ChIP-seq reveal the binding sites of lactylated histone H3. (**A**) Comparative analysis of lactylated histone H3 binding site enrichment at transcription start site (TSS) regions between the control group and the lactic acid treated group (20mM, 48 h); (**B**) Examination of the distribution of lactylated histone H3 binding sites across various genomic elements in control and lactic acid treatment groups; (**C**) Consistency analysis of biological replicates; (**D**) Venn diagram illustrating the overlap of antibody binding sites before and after lactic acid treatment; (**E**) Identification of genes associated with melanin synthesis among lactylated histone H3 binding sites; (**F**) Analysis of the binding of lactylated histone H3 at the TSS region of Tyrp1 gene before and after lactic acid treatment. “Con” denotes the control group, and “LA” denotes the lactic acid treated group.

Utilizing ChIP-seq, we investigated the genome-wide binding sites of lactylated histone H3 both before and after lactic acid treatment. Our findings revealed that lactylated histone H3 exhibited significant enrichment at transcription start site (TSS) regions (Fig. 4A). Notably, enrichment at promoter regions increased from 15.5% in the control group to 18.34% in the lactic acid treated group (Fig. 4B). The biological replicates demonstrated high consistency (Fig. 4C).

Our investigation revealed notable differences in the binding sites of lactylated histone H3 before and after lactic acid treatment, with only approximately 10% of the binding sites overlapping between the two groups (Fig. 4D). STRING analysis identified that genes involved in melanin synthesis were among the targets of lactylated H3 binding (Fig. 4E). A detailed analysis indicated that, in the control group, the TSS region of Tyrp1 contain two significant enrichment peaks for lactylated histone H3. However, following lactic acid treatment, these peaks were almost entirely abolished (Fig. 4F). This observation suggests that lactic acid treatment may markedly reduce Tyrp1 expression by inhibiting its transcription through decreased lactylation of histone H3 binding at its TSS region.

### Lactic acid treatment reduces Tyrp1 expression

**Fig. 5:**
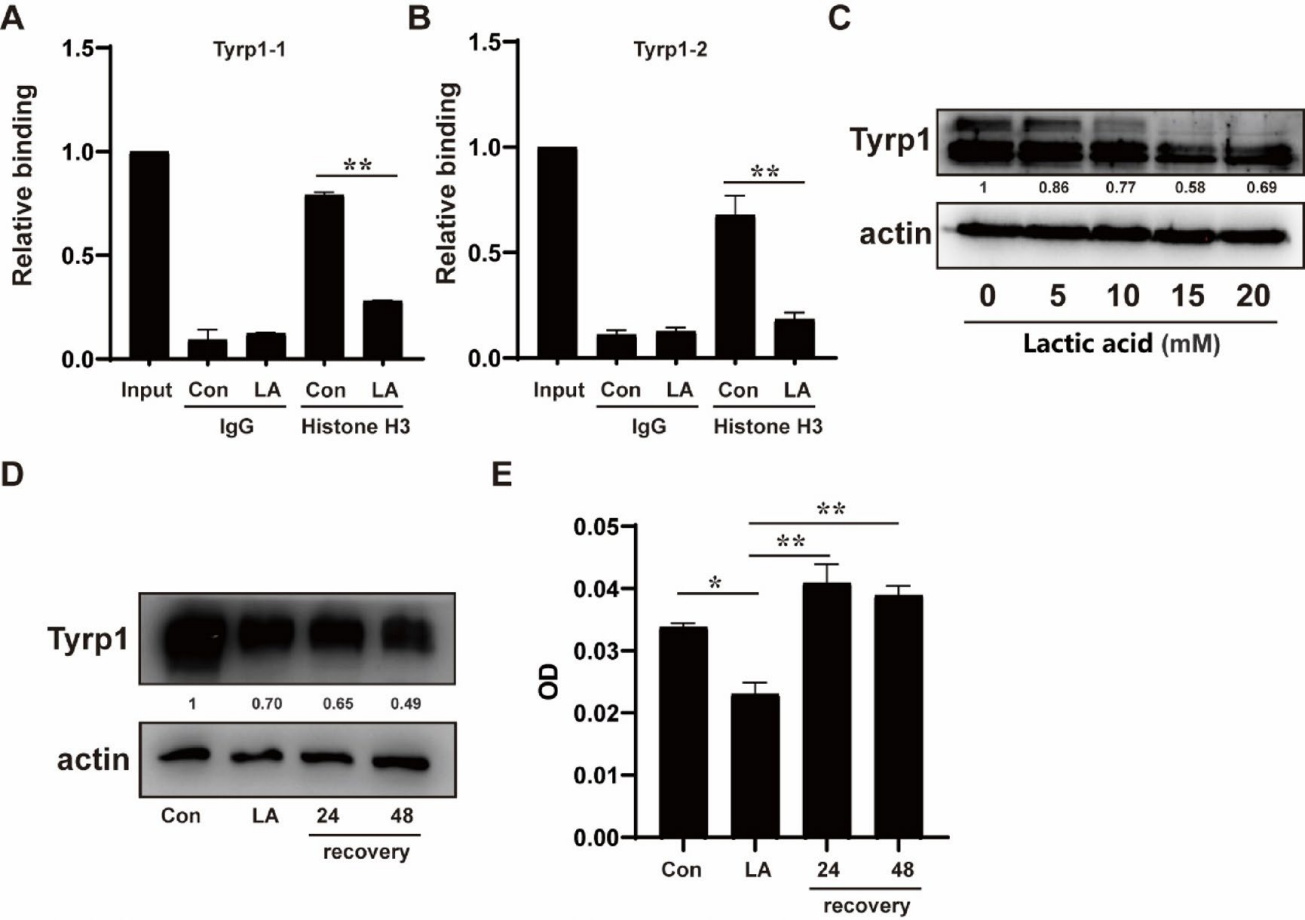
An analysis of Histone H3 binding at TSS of the Tyrp1 gene and its associated protein expression levels. (**A**,** B**) ChIP-qPCR analysis of lactylated histione H3 binding at the TSS region of the Tyrp1 gene before and after lactic acid treatment (20mM, 48 h); (**C**) Western blot analysis detects changes in Tyrp1 protein levels following treatment with 0–20 mM lactic acid for 48 h. (**D**) B16 cells were treated with 20 mM lactic acid for 48 h, followed by replacement with fresh medium to allow recovery for 24 and 48 h; (**E**) Melanin content in B16 cells was measured at each time point during the recovery experiment. **p < 0.01. “Con” for control and “LA” for lactic acid.

Additionally, we validated the binding of lactylated H3 protein in the TSS region of the Tyrp1 gene. The binding of lactylated H3 protein in TSS of Tyrp1 was abolished after the treatment of lactic acid (Fig. 5A, B). Consistently, lactic acid treatment resulted in marked decreases in protein level of Tyrp1 (Fig. 5C). In the recovery experiments following lactic acid treatment, we did not observe an increase in intracellular Tyrp1 protein levels within 48 h (Fig. 5D). However, upon returning to normal culture conditions, a significant rebound in melanin content was detected in the cells, though this recovery did not exhibit a time-dependent pattern of progressive elevation (Fig. 5E).

## Discussion

Melanin synthesis is governed by a variety of mechanisms, including the direct inhibition of tyrosinase, modulation of ROS, and suppression of melanogenic transcription factors such as MITF and SOX10^15^. Recent evidence underscores the role of epigenetic regulation in this process, with histone acetylation being shown to enhance melanocyte proliferation and metabolic activity^16^. Notably, H3K27ac is prominently associated with melanocyte enhancers^17^, and genes related to melanogenesis exhibit higher levels of H3K27ac within their gene bodies compared to those associated with the cell cycle^18^. Furthermore, the inhibition of histone deacetylases (HDACs) leads to increased melanin accumulation^19^. Suggesting that epigenetic modifications drive the transcriptional activation of genes involve in melanogenic pathways.

Histone lactylation represents the most abundant form among all known lactylatable proteins^20^. Studies demonstrate that lactic acid treatment significantly upregulates protein lactylation, which in turn promotes gene transcription or alters protein conformation and activity^21^. Our proteomic profiling demonstrates that lactic acid treatment induces dynamic changes in the lactylation landscape. As illustrated in Fig. 3, a marked difference in lactylated protein species was observed between pre- and post-treatment groups. Lactic acid not only alters cellular metabolic profiles but also modulates regulatory mechanisms of protein lactylation, it is likely mediated by lactylation writers and erasers. Genes as AARS1^12^ and EP300^22^ have been implicated in direct write in lactylation modification. However, as research in this field remains in a nascent stage, we have not yet been able to confirm whether the writer or eraser predominantly drives the differential lactylated protein profiles following lactic acid treatment.

Lactylated histones have been shown to facilitate gene transcription. In macrophages, the dynamic alterations in histone lactylation are intricately associated with the regulation of gene expression^9^. Moreover, lactylation is closely associated with tumorigenesis and cancer progression. Research indicates that under hypoxic conditions, lactic acid can induce histone H3K9 lactylation, which enhances the transcription of LAMC2, thereby facilitating the invasion of esophageal squamous cell carcinoma^23^. Similarly, histone H3K18 lactylation has been observed to elevate the transcriptional activity of the TPI1 gene, thereby accelerating the progression of osteoarthritis^24^. However, our research indicate that lactic acid treatment induces dynamic reprogramming of histone H3 lactylation: Although global histone H3K18 binding in promoter regions increases, its binding sites exhibit spatially specific redistribution, with only 10% of loci overlapping. Within the TSS of the Tyrp1 gene, this reprogramming is evidenced by the epigenetic silencing of two key binding sites located within the TSS region (Fig. 4), leading to a significant decrease in protein expression levels. This phenomenon has similarly been documented in research concerning embryonic development in murine models^25^. The findings indicate that: (1) genes regulation through lactylation demonstrates a site-selective characteristic, as opposed to simply changes in the abundance of modifications; (2) the dynamic redistribution of binding sites within promoter regions is directly associated with the transcriptional repression of downstream genes.

Tyrp1 is a key enzyme in the melanin synthesis pathway, primarily involved in the formation of eumelanin (brown-black melanin)^26^. It can form a complex with TYR and DCT, regulating the stability and activity of these enzymes^27^. The absence of TYRP1 significantly reduces eumelanin production, resulting in the inability of melanosomes to produce normal brown-black melanin. Our experimental data show that when cells recovered from a lactic acid containing environment (24 and 48 h), we did not observe restoration of Tyrp1 protein expression levels (Fig. 5D). We propose the following explanations for this phenomenon: (1) Lactic acid mediated inhibition of melanin synthesis likely involves multiple mechanisms, including pH modulation, suppression of TYR protein activity^3^, and the possibility in regulation of cellular autophagy^28,29^. Among these, epigenetic regulation (histone lactylation) plays a significant but non-exclusive role; (2) Removal of lactic acid alleviates melanogenesis suppression, but the pre-existing lactylation modification patterns may persist transiently, preventing rapid reversal at sites strongly affected by lactylation, similar phenomena are commonly observed in compound-induced epigenetic modifications^30–32^; (3) Despite the lack of Tyrp1 recovery, other proteins of melanin synthesis pathway may have partially regained expression, enabling increased melanin production. However, melanin accumulation plateaus between 24 and 48 h, suggesting a bottleneck related to lactylation and TYRP1 expression.

In conclusion, our research primary elucidates the role of Histone lactylation in regulating melanin synthesis. Nonetheless, several pertinent questions need to further investigation: (1) Does the lactylation of non-histone proteins influence melanin synthesis? (2) Is there a cross-talk between lactylation and other epigenetic regulatory mechanisms? Addressing these questions in future studies will deepen our understanding of the complex role of lactic acid in the fields of epigenetics and dermatology.

## Electronic supplementary material

Below is the link to the electronic supplementary material.

Supplementary Material 1

## Data Availability

The data that support the findings of this study are available from the corresponding author upon reasonable request. Data of proteomics could be found at PRIDE (access number: PXD060840, https://www.ebi.ac.uk/pride/archive/projects/PXD060840).
