# Supplementary figures and images for: Lactic acid inhibits melanin synthesis by regulating histone H3 lactylation and suppressing tyrp1 transcription in B16 melanoma cells
Source: Sci Rep. 2025 Jul 16;15:25816. doi: 10.1038/s41598-025-04225-8 (PMC12267477; doi:10.1038/s41598-025-04225-8)

## Slide 1
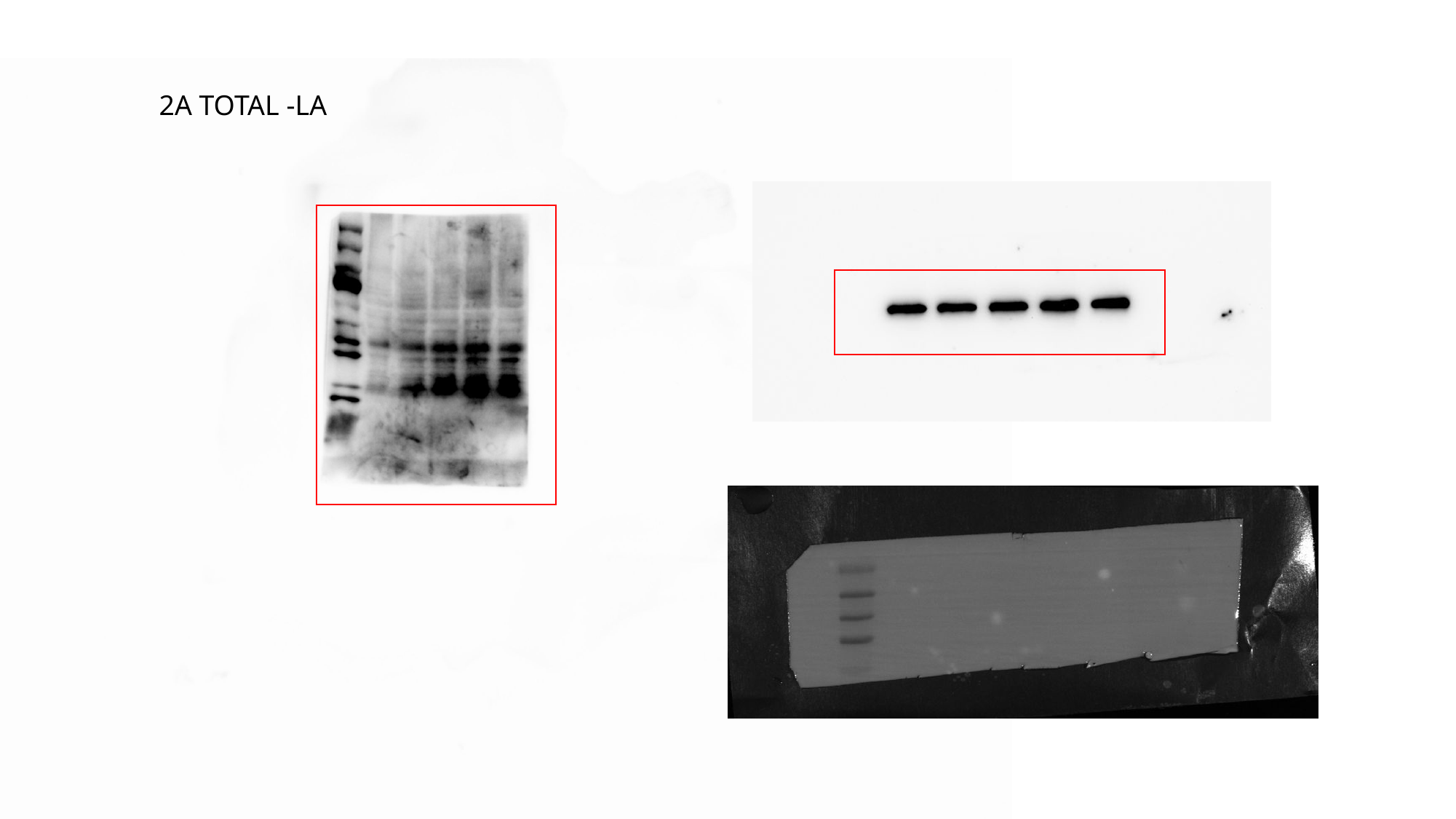

2A TOTAL -LA

## Slide 2
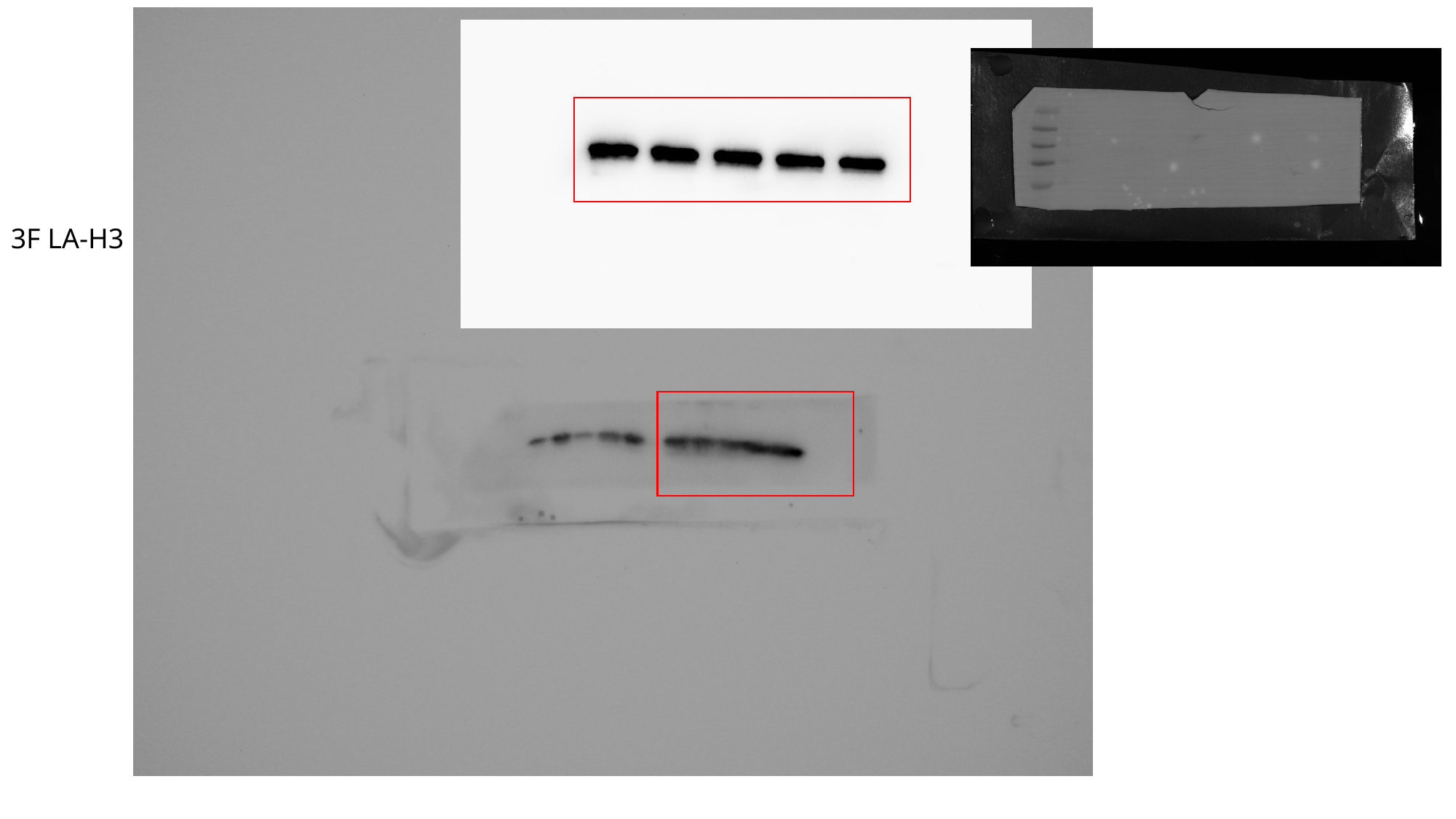

#
3F LA-H3

## Slide 3
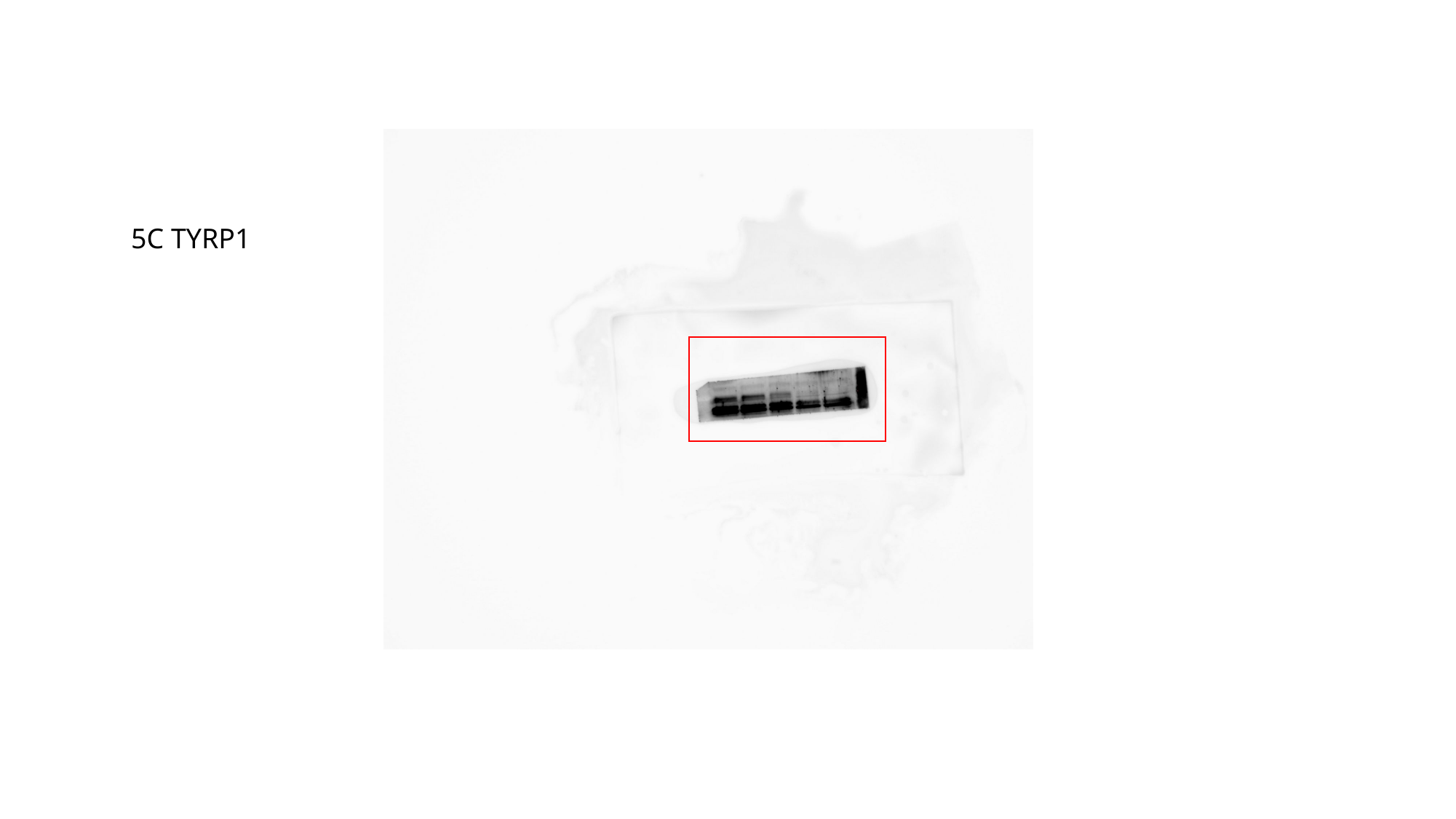

#
5C TYRP1

## Slide 4
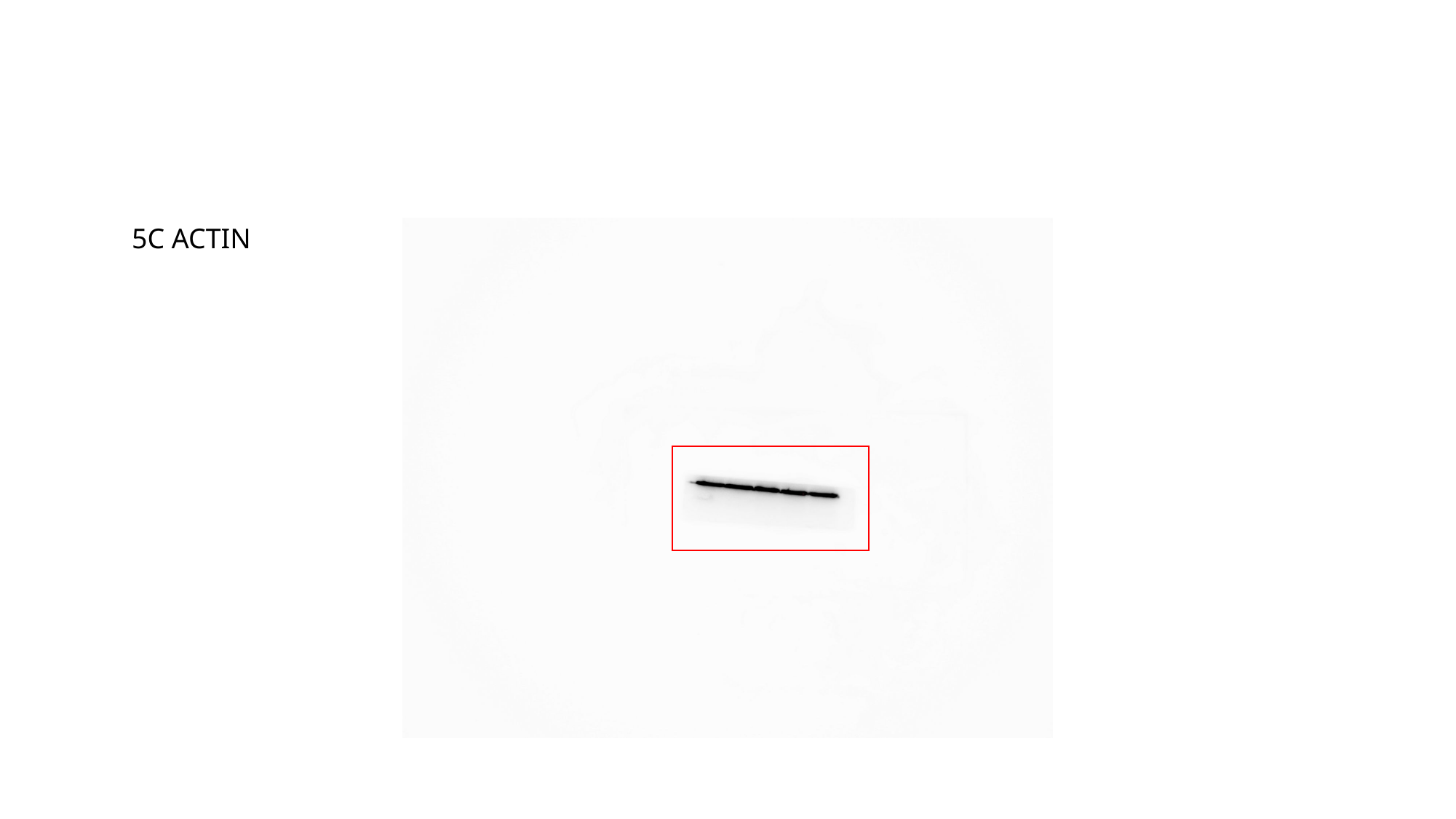

#
5C ACTIN

## Slide 5
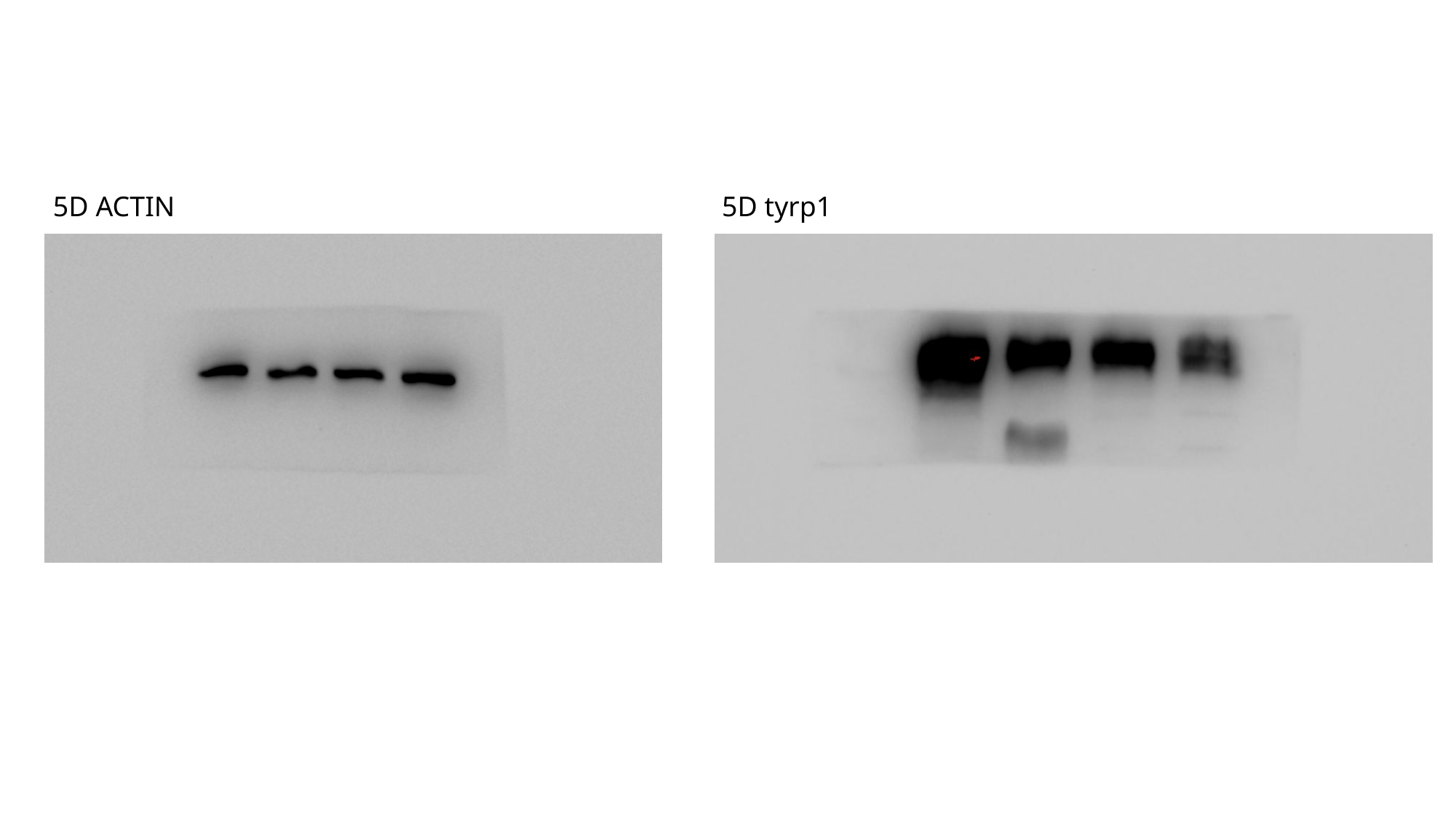

5D ACTIN
5D tyrp1

Supplement: Supplementary file 1 — Supplementary Material 1 [file 41598_2025_4225_MOESM1_ESM.pptx]
